# Moderate-to-vigorous Physical Activity and Sedentary Behavior Are Independently Associated With Renal Function: A Cross-sectional Study

**DOI:** 10.2188/jea.JE20210155

**Published:** 2023-06-05

**Authors:** Megumi Hara, Yuichiro Nishida, Keitaro Tanaka, Chisato Shimanoe, Kayoko Koga, Takuma Furukawa, Yasuki Higaki, Koichi Shinchi, Hiroaki Ikezaki, Masayuki Murata, Kenji Takeuchi, Takashi Tamura, Asahi Hishida, Mineko Tsukamoto, Yuka Kadomatsu, Keitaro Matsuo, Isao Oze, Haruo Mikami, Miho Kusakabe, Toshiro Takezaki, Rie Ibusuki, Sadao Suzuki, Hiroko Nakagawa-Senda, Daisuke Matsui, Teruhide Koyama, Kiyonori Kuriki, Naoyuki Takashima, Yasuyuki Nakamura, Kokichi Arisawa, Sakurako Katsuura-Kamano, Kenji Wakai

**Affiliations:** 1 Department of Preventive Medicine, Faculty of Medicine, Saga University, Saga, Japan; 2 Clinical Research Center, Saga University Hospital, Saga, Japan; 3 Laboratory of Exercise Physiology, Faculty of Sports and Health Science, Fukuoka University, Fukuoka, Japan; 4 Division of International Health and Nursing, Faculty of Medicine, Saga University, Saga, Japan; 5 Department of Comprehensive General Internal Medicine, Kyushu University Graduate School of Medical Sciences Faculty of Medical Sciences, Fukuoka, Japan; 6 Department of General Internal Medicine, Kyushu University Hospital, Fukuoka, Japan; 7 Department of Preventive Medicine, Nagoya University Graduate School of Medicine, Nagoya, Japan; 8 Division of Cancer Epidemiology and Prevention, Aichi Cancer Center Research Institute, Nagoya, Japan; 9 Division of Cancer Epidemiology, Nagoya University Graduate School of Medicine, Nagoya, Japan; 10 Cancer Prevention Center, Chiba Cancer Center Research Institute, Chiba, Japan; 11 Department of International Island and Community Medicine, Kagoshima University Graduate School of Medical and Dental Sciences, Kagoshima, Japan; 12 Department of Public Health, Nagoya City University Graduate School of Medical Sciences, Nagoya, Japan; 13 Department of Epidemiology for Community Health and Medicine, Kyoto Prefectural University of Medicine, Kyoto, Japan; 14 Laboratory of Public Health, Division of Nutritional Sciences, School of Food and Nutritional Sciences, University of Shizuoka, Shizuoka, Japan; 15 Department of Public Health, Faculty of Medicine, Kindai University, Osaka, Japan; 16 Department of Public Health, Shiga University of Medical Science, Shiga, Japan; 17 Yamashina Racto Clinic and Medical Examination Center, Kyoto, Japan; 18 Department of Preventive Medicine, Tokushima University Graduate School of Biomedical Sciences, Tokushima, Japan

**Keywords:** glomerular filtration rate, chronic kidney disease, isotemporal substitution model, physical activity, sedentary

## Abstract

**Background:**

Little is known about whether insufficient moderate-to-vigorous physical activity (MVPA) and longer sedentary behavior (SB) are independently associated with estimated glomerular filtration rate (eGFR) and chronic kidney disease (CKD), whether they interact with known risk factors for CKD, and the effect of replacing sedentary time with an equivalent duration of physical activity on kidney function.

**Methods:**

We examined the cross-sectional association of MVPA and SB with eGFR and CKD in 66,603 Japanese cohort study in 14 areas from 2004 to 2013. MVPA and SB were estimated using a self-reported questionnaire, and CKD was defined as eGFR <60 mL/min/1.73 m^2^. Multiple linear regression analyses, logistic regression analyses, and an isotemporal substitution model were applied.

**Results:**

After adjusting for potential confounders, higher MVPA and longer SB were independently associated with higher eGFR (*P* for trend MVPA <0.0001) and lower eGFR (*P* for trend SB <0.0001), and a lower odds ratio (OR) of CKD (adjusted OR of MVPA ≥20 MET·h/day, 0.76; 95% confidence interval [CI], 0.68–0.85 compared to MVPA <5 MET·h/day) and a higher OR of CKD (adjusted OR of SB ≥16 h/day, 1.81; 95% CI, 1.52–2.15 compared to SB <7 h/day), respectively. The negative association between MVPA and CKD was stronger in men, and significant interactions between sex and MVPA were detected. Replacing 1 hour of SB with 1 hour of physical activity was associated with about 3 to 4% lower OR of CKD.

**Conclusion:**

These findings indicate that replacing SB with physical activity may benefit kidney function, especially in men, adding to the possible evidence on CKD prevention.

## INTRODUCTION

The global prevalence of chronic kidney disease (CKD) has markedly increased in recent decades due to population aging, with the number of CKD patients of all stages reaching almost 700 million in 2017.^1^ According to the Global Burden of Disease, Injuries, and Risk Factors Study, impaired fasting plasma glucose, high blood pressure, and high body mass index (BMI) are leading risk factors for CKD.^1^ Additionally, epidemiological studies have indicated that age, male sex, lower education level, lower socioeconomic status, alcohol consumption, and lower dietary quality are independent risk factors for CKD in developed countries.^2^^–^^4^ Moreover, several recent studies have reported that lower moderate-to-vigorous physical activity (MVPA) and longer sedentary behavior (SB), risk factors for obesity, diabetes, and hypertension, are independently associated with the development of CKD.^5^^–^^7^ However, the results from some studies have been inconsistent due to differences in the measurement and analysis of physical activity and SB.^2^^,^^4^^,^^8^^,^^9^ Furthermore, the interaction of physical activity and SB with known risk factors for kidney function decline has not been fully evaluated.

Regarding physical activity, most intervention studies have used exercise programs, which are planned, structured, repetitive, and usually intended to enhance or maintain physical fitness, to improve both renal and physical function among CKD or dialysis patients.^10^^–^^12^ A meta-analysis of 13 randomized controlled trials that examined exercise therapy, including aerobic and resistance exercise, among 421 non-dialysis CKD patients showed a benefit of increasing the estimated glomerular filtration rate (eGFR).^13^ In contrast, interventions involving modification of physical activity and SB are limited even among CKD and dialysis patients, and the benefits for renal outcome have been inconclusive.^12^^,^^14^^,^^15^ This may be due to insufficient evidence indicating which daily life activity modifications actually improve kidney function in the early stages of CKD and in the general population.

In the past decade, isotemporal substitution approaches, which are used to evaluate the effect of replacing daily SB with an equivalent duration of physical activity on health, have been used to improve various outcomes.^16^ Several studies have explored the relationships between sedentary time allocation and health outcomes, such as weight change,^16^ cardiovascular disease biomarkers,^17^ cardiometabolic health,^18^ depression,^19^ mortality,^20^^,^^21^ and mortality among CKD patients.^22^ A recent study by Kosaki et al measured physical activity using a uniaxial accelerometer and reported that replacing SB with MVPA was beneficially associated with renal function in older adults.^23^ While their study design and investigation were excellent, the sample size was small, so further investigation is required to confirm the reproducibility of their results.

We investigated whether MVPA and SB are independently associated with eGFR and CKD and examined the interaction between MVPA and SB with potential risk factors for CKD among the Japanese general population. In addition, we used the isotemporal substitution approach to evaluate the effect of replacing sedentary time with an equivalent duration of physical activity on kidney function.

## METHODS

### Study population

We used data from the Japan Multi-Institutional Collaborative Cohort (J-MICC) Study. The details and rationale of the J-MICC study have been described elsewhere.^24^ Briefly, the study focuses on interactions between genetic and lifestyle factors for lifestyle-related diseases, the main one being cancer. The J-MICC study consists of 14 areas throughout Japan, including Chiba, Shizuoka-Sakuragaoka, Okazaki, Aichi, Shizuoka, Daiko, Iga, Takashima, Kyoto, Tokushima, Saga, Kagoshima, Fukuoka and the KOPS (Kyushu and Okinawa Population Study) area. Eligible participants were residents in the community, health-checkup examinees, and visitors to a cancer hospital. Participants provided written informed consent after receiving an explanation of the study purpose and contents and the conditions of cooperation in the study. They completed a self-administered questionnaire on lifestyle and medical information, and donated blood samples. The study protocol was approved by the Ethics Committees of Nagoya University Graduate School of Medicine (approval No. 2010-0939-7) and each participating institute.

A total of 92,640 participants were recruited between 2004 and 2013 (dataset version 20180602). Of the total participants, we excluded 17,196 from two areas (Chiba and Aichi) from this study because of the absence of data on creatinine. Among the remaining 75,444 participants, we excluded 8,841 with one or more of the following: no serum creatinine data (*n* = 2,936); serum creatinine <0.2 mg/dL (*n* = 17); and self-reported diseases, such as renal failure (*n* = 53), liver cirrhosis (*n* = 172), cancer (*n* = 3,016), coronary heart disease (*n* = 2,045), and cerebrovascular disease (*n* = 1,288), which may affect physical activity. Thus, the present cross-sectional study included 66,603 subjects (29,507 men and 37,096 women) for analysis of MVPA. For the evaluation of SB, we excluded 2,569 subjects who did not have available SB data. Thus, 64,034 subjects (28,175 men and 35,859 women) were included in the analysis of SB.

### Assessment of MVPA and SB

MVPA corresponding to an activity intensity ≥3 metabolic equivalents (METs) was calculated as the sum of daily life physical activity and leisure time physical activity, as assessed using a self-administered physical activity questionnaire (PAQ). This PAQ was originally developed as part of the baseline questionnaire in the J-MICC Study.^25^^–^^27^ Although it has not been validated, this PAQ has a similar structure to questionnaires that have previously been confirmed to be valid and reproducible.^28^ Further, the purpose of this PAQ was to estimate the average habitual total and domain-specific physical activity in MET-hours per day over a 1 year period. One MET was defined as an oxygen consumption of 3.5 mL/kg weight/min, which represents the average energy expenditure at rest.^29^ For daily life physical activity, the PAQ comprised four behaviors ranked according to activity level: hard labor (allocated 4.5 METs), walking (3.3 METs), standing (2.0 METs), and SB (1.5 METs). Time spent per day on each activity was categorized into one of the following eight categories (assigned average hours per day): none (0), <1 (0.5), 1 to <3 (2), 3 to <5 (4), 5 to <7 (6), 7 to <9 (8), 9 to <11 (10), and ≥11 (12) hours per day. We calculated daily hours of SB by subtracting the sum of time spent sleeping, standing, walking, and performing hard labor from 24 hours. Daily life physical activity was estimated by multiplying the amount of time spent walking and engaged in physical hard labor daily by their assigned MET intensities.

For leisure time physical activity, the PAQ was similar to a short format of the International Physical Activity Questionnaire (IPAQ),^30^^,^^31^ except all bouts of physical activity were included independent of duration. Our PAQ includes all PA, while the IPAQ does not include PA those lasting <10 minutes. Activity was categorized into three levels: vigorous (eg, marathon running and competitive sports, allocated 8.0 METs), moderate (eg, light jogging and swimming, allocated 4.0 METs), and light (eg, walking and hiking, allocated 3.3 METs), as we described previously.^32^ The frequency categories (assigned average times per day) were almost none (0), 1–3 times/month (0.1), 1–2 times/week (0.2), 3–4 times/week (0.5), and 5–6 times/week (0.8). The average duration categories (assigned average hours per activity) were <30 minutes (0.3), 30 minutes to <1 hour (0.8), 1 to <2 hours (1.5), 2 to <3 hours (2.5), 3 to <4 hours (3.5), and ≥4 hours (4.5). The MET·h/day of leisure time physical activity for each category of intensity was calculated by multiplying the daily frequency, duration, and intensity of leisure time physical activity. Thereafter, MVPA (MET·h/day) was estimated by summing the daily life physical activity and leisure time physical activity.

### Questionnaire and anthropometric and biochemical measurements

At the time of enrollment, a self-administered questionnaire was used to obtain participants’ lifestyle information, including sex, age, smoking habit, alcohol consumption, daily coffee consumption, dietary behavior over the past year, medical history, perceived stress status, and sleep duration, as well as activity in daily life and leisure time.^32^ Smoking status was classified as current, former or never. Alcohol consumption (g/day) was estimated according to average intake frequency and quantity by beverage type. Dietary intake of energy (kcal/day) was estimated using a validated food frequency questionnaire containing 47 food items.^33^ Data from anthropometric measurements, including height (to the nearest 0.1 cm) and body weight (to the nearest 0.1 kilogram), systolic blood pressure (SBP; mm Hg), serum total cholesterol (mg/dL), serum creatinine (mg/dL), and hemoglobin A1c (%), were obtained. BMI was calculated using the following formula: body weight (kg)/(height [m])^2^. History of hypertension was defined as SBP >140 mm Hg and/or diastolic blood pressure (DBP) >90 mm Hg or use of anti-hypertensive medication. History of diabetes mellitus was defined as a fasting serum glucose level ≥126 mg/dL, hemoglobin A1c level ≥6.9% (National Glycohemoglobin Standardization Program), or use of anti-diabetic medication. History of dyslipidemia was defined as fasting serum total cholesterol level ≥220 mg/dL and/or fasting low density lipoprotein cholesterol level ≥140 mg/dL and/or use of anti-dyslipidemic medication.

### eGFR and definition of CKD

eGFR was calculated based on participants’ serum creatinine, age, and sex according to the Japanese Society of Nephrology,^34^ using the following formula: eGFR (mL/min/1.73 m^2^) = 194 × serum creatinine (mg/dL)^−1.094^ × age^−0.287^ (× 0.739 if female)^34^ We defined CKD as eGFR <60 mL/min/1.73 m^2^, which is indicative of stage G3a CKD or higher,^35^ because we did not have urine data.

### Statistical analysis

Continuous variables are expressed as mean and standard deviation, and categorical variables as number and proportion (%). For analysis of MVPA, 66,603 subjects were divided into four categories of MVPA (<5, ≥5 and <10, ≥10 and <20, and ≥20 MET·h/day), while 64,034 subjects were divided into five categories of sedentary time (<3, ≥3 and <5, ≥5 and <7, ≥7 and <9, and ≥9 hours/day) for analysis of SB. The cross-sectional association of MVPA and SB categories with eGFR (mL/min/1.73 m^2^) and CKD was evaluated using multiple linear regression analyses and logistic regression analyses. We adjusted for the following covariates to examine whether they affect the association of MVPA and SB with kidney function: model 1, age, sex, and study sites; model 2, further adjusted for health-related behaviors such as alcohol consumption (g/day), current smoking (yes or no) and daily coffee consumption (yes or no); model 3, further adjusted for history of hypertension, hyperlipidemia, and diabetes (yes or no), and BMI (kg/m^2^, quartiles), factors that may confound but also mediate associations of MVPA and SB with kidney function; model 4, further adjusted for SB (h/day) for MVPA analysis and MVPA (MET·h/day) for SB analysis. Potential confounders and mediators were selected based on associations reported in the literature^2^^–^^6^ or a significant univariate correlation. The linear trend for risk was evaluated relative to the level of MVPA (as a continuous variable for 1 MET·h/day) and SB (as a continuous variable for 1 hour). To examine interactions between MVPA or SB and CKD risk, multiplicative interaction terms were added to the model. We also conducted stratified analyses according to baseline characteristics that showed a significant interaction. Additionally, 64,034 subjects were divided into two strata based on MVPA (<10 and ≥10 MET·h/day) and five categories of sedentary time (<3, ≥3 and <5, ≥5 and <7, ≥7 and <9, and ≥9 hours/day) to examine the CKD risk according to SB stratified by MVPA.

In addition, we used two models, a single factor model and an isotemporal substitution model, to assess the cross-sectional associations of daily life physical activity, including SB, standing, walking, and hard labor, with eGFR and CKD using multiple linear regression and logistic regression analyses. We used 1 hour/day as the unit for daily life physical activity and defined waking hours as the sum of hours spent performing each behavior per day. Details of these models are described elsewhere.^36^^,^^37^ The single factor model assessed each behavior component separately and was only adjusted for waking hours and the confounders listed for model 3 above. The isotemporal substitution model specifies a “target” behavior that is to be replaced with a behavior of interest, while holding total waking hours constant. This model can also be expressed by omitting the target behavior from the model and adding total waking hours. For example, the coefficient can be interpreted as the effect of replacing SB with the same duration of standing, since walking, hard labor, and total waking hours are held constant.

In the multivariable models, we calculated variance inflation factors (VIFs) to check for multicollinearity among the independent variables. All VIFs in the isotemporal substitution model were below 5, the pre-determined threshold value. In addition, correlation coefficients between variables did not exceed 0.7 using the isotemporal substitution model, and the sample size was sufficient. Thus, the analyzed data met the theoretical assumptions of the isotemporal substitution model.

All analyses were performed using the SAS statistical software package (Ver. 9.4 for Windows; SAS Institute, Cary, NC, USA). A *P* value of less than 0.05 was considered statistically significant.

## RESULTS

Among the 92,640 subjects who participated in the baseline survey, 66,603 subjects were included in this analysis. The baseline characteristics of the 66,603 included subjects differed from those of the 26,037 excluded subjects (eTable 1). Briefly, all baseline characteristics except sex; daily coffee consumption; leisure time physical activity level; and sleeping, walking, and standing hours differed between the two groups. These differences may have arisen by excluding subjects who had self-reported disease.

The characteristics of the 66,603 participants (29,507 men and 37,096 women) according to eGFR categories are shown in Table 1. The number and proportion of missing data are indicated in the footnote. In general, the majority of participants with lower eGFR were men, older, had lower alcohol consumption, lower coffee consumption, higher energy intake, took anti-hypertensives, anti-dyslipidemics, and anti-hyperglycemics, had higher SBP, lower MVPA, longer sleep and SB hours, and shorter waking hours. Additional analysis stratified by MVPA and SB is shown in eTable 2. Participants with lower MVPA were younger; had higher education levels; higher coffee consumption; took anti-dyslipidemics; had lower eGFR; higher CKD; lower leisure time physical activity; and lower hard labor, walking, and standing hours. Participants with longer SB were men; younger; had higher education levels; higher coffee consumption; lower eGFR; higher CKD; lower MVPA and leisure time physical activity; and lower hard labor, walking, and standing hours.

**Table 1.** Characteristics of study participants at the baseline survey of the Japan Multi Institutional Collaborative Cohort study by estimated glomerular filtration rate category (*n* = 66,603)

Characteristic	eGFR (mL/min per 1.73 m^2^)									

	≥90 (*n* = 12,970)		60 to 89 (*n* = 48,267)		45 to 59 (*n* = 4,971)		30 to 44 (*n* = 316)		<30 (*n* = 79)	
Men	4,492	(34.6)	22,102	(45.8)	2,672	(53.8)	195	(61.7)	46	(58.2)
Age, years	51.4	[9.7]	55.4	[9.0]	60.2	[7.0]	61.5	[6.7]	61.4	[6.7]
Education ≤12 years	5,096	(39.3)	17,501	(33.4)	1,673	(33.7)	120	(29.0)	21	(26.6)
Current smoker	2,646	(20.4)	8,264	(15.8)	606	(12.2)	46	(11.1)	19	(24.1)
Alcohol consumption, g/day	6.5	[15.0]	6.8	[14.3]	6.4	[13.1]	6.2	[13.1]	2.7	[8.3]
Drink coffee everyday	7,192	(55.5)	26,809	(51.2)	2,631	(52.9)	137	(33.1)	26	(32.9)
Total energy intake, kcal/day	1,689	[394]	1,712	[378]	1,728	[360]	1,737	[335.7]	1,671	[354.3]
Taking antihypertensives	1,420	(10.9)	7,137	(13.6)	1,302	(26.2)	177	(42.8)	58	(73.4)
Taking antidislipidemics	870	(6.7)	4,355	(8.3)	4,218	(84.9)	242	(58.5)	55	(69.6)
Taking antihyperglycemics	514	(4.0)	1,516	(2.9)	236	(4.7)	48	(11.6)	15	(19.0)
BMI, kg/m^2^	22.7	[3.5]	23.0	[3.2]	23.7	[3.2]	24.3	[3.4]	22.9	[3.9]
SBP, mm Hg	125.3	[19.9]	127.6	[19.8]	131.8	[20.2]	137.9	[22.4]	141.9	[24.3]
Total cholesterol, mg/dL	206.5	[35.5]	211.7	[34.3]	215.9	[34.8]	211.1	[41.4]	196.4	[39.9]
HbA1c	5.2	[0.9]	5.2	[0.6]	5.2	[0.6]	5.5	[1.2]	5.3	[0.7]
Creatinine, mg/dL	0.6	[0.1]	0.7	[0.1]	1.0	[0.1]	1.3	[0.2]	4.7	[3.6]
Total physical activity, MET·h/day	16.3	[14.5]	15.2	[13.6]	14.7	[13.4]	15.0	[14.0]	14.0	[16.1]
Leisure time physical activity, MET·h/day	1.3	[2.1]	1.7	[2.5]	2.1	[2.8]	1.9	[2.4]	1.5	[2.2]
Activities performed in daily life	(*n* = 12,273)		(*n* = 46,559)		(*n* = 4,822)		(*n* = 305)		(*n* = 75)	
Sleeping, h/day	6.6	[1.0]	6.6	[1.0]	6.7	[1.0]	6.8	[1.1]	7.0	[1.1]
Hard labor, h/day	1.1	[1.9]	1.0	[1.8]	1.0	[1.8]	1.0	[1.8]	0.9	[1.7]
Walking, h/day	2.4	[2.0]	2.2	[2.0]	2.1	[1.9]	2.1	[1.8]	1.8	[2.0]
Standing, h/day	3.9	[2.7]	3.4	[2.6]	3.0	[2.5]	2.8	[2.4]	2.9	[3.1]
Sedentary behavior, h/day	10.1	[4.4]	10.7	[4.3]	11.2	[4.3]	11.3	[4.4]	11.4	[5.0]

BMI, body mass index; eGFR, estimated glomerular filtration rate; METs, metabolic equivalents; SBP, systolic blood pressure.

Data are presented as mean [standard deviation] or number (percentage).

Note: Missing data on current smoking (*n* = 309, 0.5%), alcohol consumption (*n* = 1,247, 1.9%), everyday coffee drinking (*n* = 7, 0.01%), take antihypertensives (*n* = 51, 0.08%), antidyslipidemics (*n* = 51, 0.08%), and antihyperglycemics (*n* = 51, 0.08%).

In regression analysis, lower MVPA and longer SB were both associated with lower eGFR levels (Table 2). While adjustment for age, sex, study area (model 1), and baseline characteristics (models 2 and 3) slightly attenuated these correlations, they remained significant (Table 2). In the multivariable model including both MVPA and SB, these correlations remained statistically significant, indicating that MVPA and SB were independently associated with eGFR (Table 2, model 4). Analysis stratified by MVPA (<10 and ≥10 MET·h/day) showed that adjusted eGFR with longer SB was similar between the MVPA groups (*P*_for interaction_ = 0.46) (eFigure 1).

**Table 2.** Adjusted mean estimated glomerular filtration rate according to moderate-to-vigorous physical activity (*n* = 66,603) and sedentary behavior (*n* = 64,034)

	Number	Crude		Model 1		Model 2		Model 3		Model 4	
				
		Mean (95% CI)		Adjusted mean (95% CI)		Adjusted mean (95% CI)		Adjusted mean (95% CI)		Adjusted mean (95% CI)	
MVPA, MET·h/day											
<5	16,672	77.9	(77.7–78.1)	77.4	(77.2–77.7)	77.5	(77.3–77.7)	77.4	(77.2–77.6)	77.5	(77.3–77.7)
≥5, <10	14,408	78.3	(78.0–78.5)	77.7	(77.5–77.9)	77.8	(77.6–78.0)	77.8	(77.6–78.0)	77.9	(77.6–78.1)
≥10, <20	17,399	78.4	(78.2–78.6)	78.6	(78.4–78.8)	78.6	(78.4–78.8)	78.6	(78.3–78.8)	78.6	(78.3–78.8)
≥20	18,124	79.3	(79.1–79.5)	80.0	(79.8–80.2)	79.9	(79.7–80.1)	79.8	(79.6–80.0)	79.7	(79.5–79.9)
	β	0.43	(0.33–0.53)	0.94	(0.84–1.03)	0.77	(0.68–0.87)	0.79	(0.69–0.87)	0.72	(0.62–0.82)
		*P*_trend_ < 0.0001		*P*_trend_ < 0.0001		*P*_trend_ < 0.0001		*P*_trend_ < 0.0001		*P*_trend_ < 0.0001	

SB, h/day											
<7	12,928	79.9	(79.7–80.2)	80.3	(80.1–80.6)	80.2	(80.0–80.4)	80.1	(79.9–80.4)	80.2	(79.8–80.5)
≥7, <10	13,350	79.2	(78.9–79.4)	79.1	(78.9–79.3)	79.1	(78.8–79.3)	79.0	(78.8–79.2)	79.0	(78.8–79.2)
≥10, <13	14,394	78.6	(78.3–78.8)	78.4	(78.2–78.6)	78.5	(78.2–78.7)	78.4	(78.2–78.6)	78.4	(78.2–78.6)
≥13, <16	15,311	77.2	(77.0–77.5)	77.1	(76.9–77.3)	77.2	(76.9–77.4)	77.1	(76.9–77.3)	77.1	(76.8–77.3)
≥16	8,051	76.3	(76.0–76.6)	76.4	(76.1–76.7)	76.5	(76.2–76.8)	76.5	(76.1–76.8)	76.4	(76.1–76.8)
	β	−0.90	(−0.99 to −0.82)	−0.99	(−1.07 to −0.91)	−0.84	(−0.93 to −0.76)	−0.86	(−0.94 to −0.77)	−0.87	(−0.99 to −0.75)
		*P*_trend_ < 0.0001		*P*_trend_ < 0.0001		*P*_trend_ < 0.0001		*P*_trend_ < 0.0001		*P*_trend_ < 0.0001	

CI, confidence interval; MVPA, moderate-to-vigorous physical activity; SB, sedentary behavior.

Model 1: adjusted for age, sex (for all), and study site.

Model 2: additionally adjusted for education level (≤12 years or >12 years), current smoking (yes or no), alcohol consumption (g/day) and daily coffee consumption (yes or no).

Model 3: additionally adjusted for history of hypertension (yes or no), history of hyperlipidemia (yes or no), history of diabetes (yes or no), body mass index (quartile).

Model 4: additionally adjusted for sedentary behavior (h/day) or total physical activity (MET·h/day).

**Figure 1.**
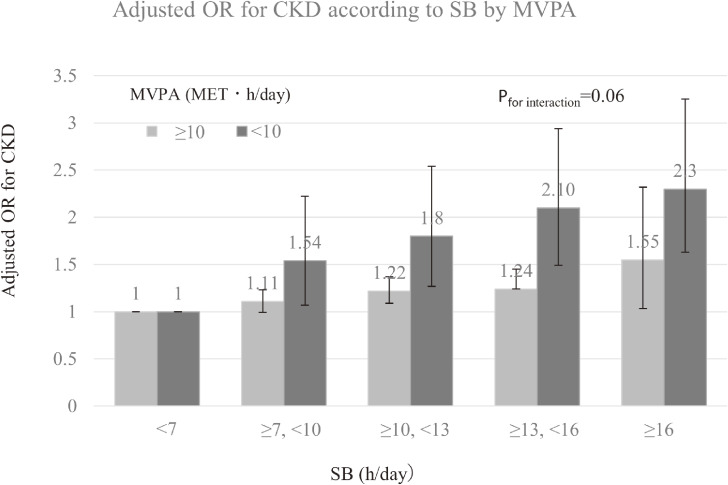
Adjusted odd ratios (ORs) for chronic kidney disease (CKD) according to sedentary behavior (SB; h/day) by moderate-to-vigorous physical activity (MVPA; MET·h/day). Adjusted variables are age, study site, education level (≤12 years or >12 years), current smoking (yes or no), alcohol consumption (g/day), daily coffee consumption (yes or no), history of hypertension (yes or no), history of hyperlipidemia (yes or no), history of diabetes (yes or no), and body mass index (quartile). Error bars indicate 95% confidence interval. *P* for trends of adjusted OR for CKD among subjects with MVPA ≥10 and <10 were <0.001 and <0.002, respectively.

Table 3 shows the association of MVPA and SB with CKD. In analysis that adjusted for age, sex, and study area, a lower odds ratio (OR) of CKD was observed in subjects with higher MVPA, while a higher OR of CKD was found in subjects with longer SB (*P*_for trend_ < 0.0001 for both) (Table 3, model 1). These associations remained significant after adjustment for health behaviors (Table 3, model 2) and comorbidities (Table 3, model 3). Compared to subjects whose MVPA was <5 MET·h/day, adjusted ORs of CKD of subjects whose MVPA was ≥10 MET·h/day and ≥20 MET·h/day were 0.83 (95% confidence interval [CI], 0.77–0.91) and 0.74 (95% CI, 0.68–0.80), respectively (Table 3, model 3). Further adjustment for SB in model 4 attenuated the trend in ORs of CKD for active MVPA, leading them to remain significant, although adjusted ORs were slightly increased (Table 3, model 4). Compared to subjects with a sedentary time of less than 7 hours/day, adjusted ORs of CKD of subjects whose sedentary time was ≥7 to <10 hours/day, ≥10 to <13 hours/day, ≥13 to <16 hours/day, and ≥16 hours/day were 1.13 (95% CI, 1.02–1.25), 1.28 (95% CI, 1.16–1.41), 1.46 (95% CI, 1.32–1.60), and 1.65 (95% CI, 1.47–1.84), respectively (Table 3, model 3). In contrast to MVPA, analysis of SB showed that fully adjusted ORs of CKD for longer sedentary time were unchanged (Table 3, model 4). Analysis stratified by MVPA (<10 and ≥10 MET·h/day) showed that CKD risk with longer SB was greater in subjects with lower MVPA (*P*_for interaction_ = 0.06) (Figure 1).

**Table 3.** Odds ratios of chronic kidney disease according to moderate-to-vigorous physical activity (*n* = 66,603) and sedentary behavior (*n* = 64,034)

MVPA (MET·h/day)	Number	eGFR <60	Crude		Model 1		Model 2		Model 3		Model 4	
		*n*	OR (95% CI)		Adjusted OR (95% CI)		Adjusted OR (95% CI)		Adjusted OR (95% CI)		Adjusted OR (95% CI)	
All (*n* = 66,603)												
<5	16,672	1,654	1	(reference)	1	(reference)	1	(reference)	1	(reference)	1	(reference)
≥5, <10	14,408	1,380	0.97	(0.89–1.05)	1.00	(0.92–1.08)	0.99	(0.91–1.08)	0.988	(0.91–1.08)	0.987	(0.91–1.08)
≥10, <20	17,399	1,622	0.91	(0.84–0.98)	**0.82**	**(0.76–0.89)**	**0.82**	**(0.75–0.89)**	**0.83**	**(0.77–0.91)**	**0.84**	**(0.77–0.91)**
≥20	18,124	1,563	**0.87**	**(0.81–0.94)**	**0.71**	**(0.65–0.77)**	**0.72**	**(0.66–0.78)**	**0.74**	**(0.68–0.80)**	**0.76**	**(0.69–0.83)**
			*P*_trend_ < 0.0001		*P*_trend_ < 0.0001		*P*_trend_ < 0.0001		*P*_trend_ < 0.0001		*P*_trend_ < 0.0001	

SB (h/day)	Number	eGFR <60	Crude		Model 1		Model 2		Model 3		Model 4	
		*n*	OR (95% CI)		Adjusted OR (95% CI)		Adjusted OR (95% CI)		Adjusted OR (95% CI)		Adjusted OR (95% CI)	

All (*n* = 64,034)												
<7	12,928	880	1	(reference)	1	(reference)	1	(reference)	1	(reference)	1	(reference)
≥7, <10	13,360	954	1.05	(0.96–1.16)	**1.13**	**(1.03–1.25)**	**1.14**	**(1.04–1.26)**	**1.13**	**(1.02–1.25)**	**1.13**	**(1.02–1.26)**
≥10, <13	14,394	1,159	**1.20**	**(1.09–1.31)**	**1.30**	**(1.18–1.43)**	**1.30**	**(1.18–1.43)**	**1.28**	**(1.16–1.41)**	**1.28**	**(1.14–1.44)**
≥13, <16	15,311	1,383	**1.36**	**(1.25–1.48)**	**1.53**	**(1.40–1.68)**	**1.50**	**(1.37–1.65)**	**1.46**	**(1.32–1.60)**	**1.46**	**(1.28–1.66)**
≥16	8,051	826	**1.57**	**(1.42–1.73)**	**1.80**	**(1.63–2.00)**	**1.72**	**(1.54–1.92)**	**1.65**	**(1.47–1.84)**	**1.65**	**(1.42–1.92)**
			*P*_trend_ < 0.0001		*P*_trend_ < 0.0001		*P*_trend_ < 0.0001		*P*_trend_ < 0.0001		*P*_trend_ < 0.0001	

CI, confidence interval; CKD, chronic kidney disease; eGFR, estimated glomerular filtration rate; MVPA, moderate-to-vigorous physical activity; OR, odds ratio; SB, sedentary behavior.

Bold values indicate *P* < 0.05.

Model 1: adjusted for age, sex (for all), and study site.

Model 2: additionally adjusted for education level (≤12 years or >12 years), current smoking (yes or no), alcohol consumption (g/day) and daily coffee consumption (yes or no).

Model 3: additionally adjusted for history of hypertension (yes or no), history of hyperlipidemia (yes or no), history of diabetes (yes or no), body mass index (quartile).

Model 4: additionally adjusted for sedentary behavior (h/day) or total physical activity (MET·h/day).

**Figure 2.**
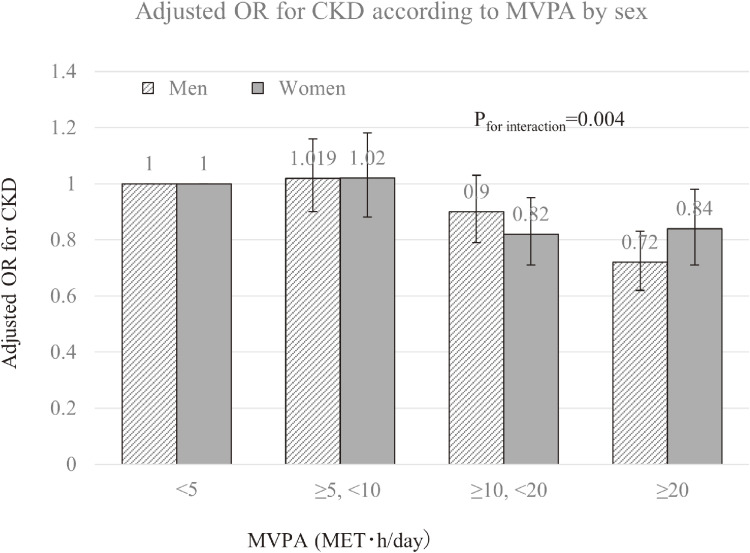
Adjusted odd ratios (ORs) for chronic kidney disease (CKD) according to moderate-to-vigorous physical activity (MVPA; MET·h/day) by sex. Adjusted variables are age, study site, education level (≤12 years or >12 years), current smoking (yes or no), alcohol consumption (g/day), daily coffee consumption (yes or no), history of hypertension (yes or no), history of hyperlipidemia (yes or no), history of diabetes (yes or no), body mass index (quartile), and sedentary behavior (hours/day). Error bars indicate 95% confidence interval. *P* for trends of adjusted OR for CKD among men and women were 0.528 and 0.038, respectively.

Additional analysis stratified by baseline characteristics showed significant interactions between sex and MVPA for CKD. The negative dose-response association between the adjusted OR of CKD and active MVPA was more evident in men than women (*P*_for interaction_ = 0.013) (Figure 2).

Table 4 shows the eGFR and CKD findings for SB. In the single factor model, sedentary time was significantly associated with lower eGFR (β = −0.31, 95% CI, −0.34 to −0.28) and higher OR of CKD (adjusted OR 1.05, 95% CI, 1.04–1.06), while time spent standing, walking, and performing hard labor was significantly positively associated with eGFR and negatively associated with OR of CKD. In the isotemporal substitution model, replacing sedentary time with standing, walking, and hard labor was significantly associated with larger eGFR values. The positive association with eGFR was greater when sedentary time was replaced with hard labor than with standing and walking. Replacing sedentary time with walking and hard labor was also significantly negatively associated with OR of CKD.

**Table 4.** Single factor model and isotemporal substitution model examining the associations of sedentary behavior, standing, walking, and hard labor with estimated glomerular filtration rate and chronic kidney disease

		Sedentary behavior	Standing	Walking	Hard labor
eGFR	Model	β (95% CI)	β (95% CI)	β (95% CI)	β (95% CI)

	Single factor model	**−0.30 (−0.33 to −0.28)**	**0.37 (0.33–0.42)**	**0.33 (0.27–0.38)**	**0.45 (0.40–0.52)**
	Isotemporal substitution model				
	Replace 1 hour of sedentary time with other behavior	Dropped	**0.31 (0.27–0.36)**	**0.21 (0.15–0.27)**	**0.39 (0.33–0.45)**
	Replace 1 hour of standing time with other behavior	**−0.31 (−0.36 to −0.27)**	Dropped	**−0.10 (−0.18 to −0.02)**	**0.08 (0.02–0.14)**
	Replace 1 hour of walking time with other behavior	**−0.21 (−0.27 to −0.15)**	**0.10 (0.02–0.18)**	Dropped	**0.18 (0.09–0.27)**
	Replace 1 hour of hard labor time with other behavior	**−0.39 (−0.45 to −0.33)**	**−0.08 (−0.16 to −0.01)**	**−0.18 (−0.27 to −0.09)**	Dropped

CKD	Model	OR (95% CI)	OR (95% CI)	OR (95% CI)	OR (95% CI)

	Single factor model	**1.04 (1.04–1.05)**	**0.95 (0.94–0.96)**	**0.95 (0.94–0.97)**	0.97 (0.94–1.00)
	Isotemporal substitution model				
	Replace 1 hour of sedentary time with other behavior	Dropped	**0.96 (0.94–0.97)**	**0.96 (0.94–0.97)**	**0.97 (0.95–0.99)**
	Replace 1 hour of standing time with other behavior	**1.05 (1.03–1.06)**	Dropped	1.02 (0.99–1.04)	1.00 (0.98–1.02)
	Replace 1 hour of walking time with other behavior	**1.03 (1.01–1.05)**	0.99 (0.96–1.01)	Dropped	0.98 (0.96–1.01)
	Replace 1 hour of hard labor time with other behavior	**1.05 (1.03–1.07)**	1.00 (0.98–1.02)	1.02 (0.99–1.04)	Dropped

CI, confidence interval; CKD, chronic kidney disease; OR, odds ratio. Bold values indicate *P* < 0.05.

Adjusted for age, sex (for all), study site, educational level (≤12 years or >12 years), current smoking (yes or no), alcohol consumption (g/day), history of hypertension (yes or no), history of hyperlipidemia (yes or no), history of diabetes (yes or no), body mass index (quartile).

## DISCUSSION

In our study of a middle-aged Japanese general population, lower MVPA and longer SB were independently negatively associated with kidney function and positively associated with OR of CKD after adjustment for other established risk factors, such as sex, age, alcohol consumption, smoking, obesity, hypertension, and diabetes, in concordance with previous studies in Western countries.^1^^–^^6^^,^^8^^,^^9^ We found that higher OR of CKD with longer SB was more evident in subjects with lower MVPA. Additionally, interactions between MVPA and sex were observed for CKD. A major finding was that replacing SB with standing, walking, and hard labor was significantly positively associated with eGFR and negatively associated with CKD. These findings suggest that modification of daily life activity has a preventive effect on kidney function decline in the general population, and the effect may be most pronounced in men.

The mechanisms underlying the associations of MVPA and SB with eGFR are multifactorial and have not been fully clarified. A study reported that inflammation, oxidative stress, and endothelial dysfunction are inversely associated with eGFR.^38^ Sedentary behavior can lead to overweight and obesity, where the accumulation of adipose tissue can induce inflammation and oxidative stress.^39^ However, physical activity has been shown to reduce inflammation and oxidative stress in both the general population and patients with CKD.^11^ We previously reported the presence of an inverse correlation between daily physical activity and levels of circulating inflammatory cytokines, including interleukins^40^ and urine 8-hydroxydeoxyguanosine, as an index of whole-body oxidative stress.^41^ Further, while prolonged SB markedly reduces micro- and macrovascular dilator function,^42^ these impairments can be improved with walking.^43^ Thus, physical activity is expected to have protective effects on renal function.

We found significant interactions between MVPA and sex for CKD in this study. Gender-dependent incidence and progression of chronic renal disease have been observed in previous epidemiological studies, which suggested that sex hormones may have an effect on renal structure and function.^44^ One study reported that estrogen has pleiotropic effects on various cell types, including renal cells, and that ovarian hormones have a protective effect against renal aging.^45^ We thus speculate that the protective effects of female hormones may explain the lower protective effects of MVPA on CKD in women.

Given that comorbidities, such as obesity, diabetes, and hyperlipidemia may affect the impact of MVPA and SB on eGFR and CKD, adjustments for these comorbidities may have resulted in over-adjustment bias. However, small changes in the regression coefficients of eGFR and OR of CKD after adjustment in model 3 suggest that these factors are not mediators, and that MVPA and SB are associated with renal function independent of these comorbidities.

Compared to individuals whose sedentary time was <7 hours, individuals whose sedentary time was ≥10 hours had about 40% higher adjusted OR of CKD. Meanwhile, isotemporal substitution analysis showed that replacing 1 hour of SB with 1 hour of standing, walking, and hard labor led to 5% lower OR of CKD and significantly higher eGFR values of 0.32 mL/min/1.73 m^2^ (95% CI, 0.27–0.37), 0.22 mL/min/1.73 m^2^ (95% CI, 0.16–0.29), and 0.42 mL/min/1.73 m^2^ (95% CI, 0.35–0.50), respectively. These results suggest that replacing SB with not only hard labor but also standing can provide a significant benefit to kidney function.

A strength of this study is that we evaluated the effect of MVPA and SB not only on CKD risk but also on eGFR in healthy individuals. Most previous studies have used data from CKD patients and evaluated CKD progression. To our knowledge, this is the largest study to examine kidney function in the general population using isotemporal substitution analysis with adjustment for multiple potential confounders. We had few missing data, and multiple imputations analysis using the chained equation method produced comparable results to those using the original dataset (data not shown).

However, this study also has several limitations. First, due to its cross-sectional nature, causal relationships of MVPA and SB with kidney function could not be evaluated in this study, although a recent prospective study revealed that a greater number of sedentary workers developed CKD than workers who stood or walked at work.^46^ Physical activity may be lower and SB may be longer in individuals with reduced eGFR with muscle loss in renal disease.^47^ To minimize the potential of reverse causality, we excluded individuals who had a history of renal disease. Second, assessment of physical activity was based on self-report, which may be associated with some degree of misclassification. However, such misclassification may be non-differential and may have little attenuation on the true associations. Consistent with our results, a recent study that assessed PA using an accelerometer found that replacing SB with MVPA may benefit renal health.^23^ Third, CKD was defined according to eGFR only, and was not linked to proteinuria or duration of kidney function decline. Because we defined CKD as eGFR <60 mL/min/1.73 m^2^, which is representative of stage 3a CKD or higher,^35^ the effect of MVPA and SB on earlier stages of CKD remains unclear. Fourth, unmeasured residual confounding must be considered when interpreting the results. For example, frailty status may be associated with both SB and renal function and may have confounded the association between these factors. Finally, selection bias may have occurred through data cleaning. Subjects included this analysis were healthier than those who were excluded. In fact, prevalence of CKD was 8.6% in this study, which is lower than that found for representative populations of Japanese in this age group.^48^^,^^49^ The healthy volunteer effect may have affected the true associations, and our results may not be generalizable to other age or ethnic groups.

### Conclusion

We found that insufficient MVPA and longer SB were independently negatively associated with eGFR and positively associated with OR of CKD in Japanese men and women. Theoretically, replacing SB with daily life activity was estimated to lead to higher eGFR and lower OR of CKD in the general population, a finding that may be most pronounced in men. A longitudinal study is needed to confirm our findings.

## SUPPLEMENTARY MATERIAL

eTable 1. Characteristics of study participants at the baseline survey of the Japan Multi-Institutional Collaborative Cohort study by inclding status (n = 92,640).

eTable 2. Characteristics of study participants at the baseline survey of the Japan Multi-Institutional Collaborative Cohort study moderate-to-vigorous physical activity (n = 66,603) and sedentary behavior (n = 64,034).

eFigure 1. Adjusted mean eGFR according to sedentary behavior (h/day) by moderate-to-vigorous physical activity (MVPA; MET·h/day)
