# Supplementary material for: Moderate-to-vigorous Physical Activity and Sedentary Behavior Are Independently Associated With Renal Function: A Cross-sectional Study
Source: J Epidemiol. 2023 Jun 5;33(6):285–93. doi: 10.2188/jea.JE20210155 (PMC10165219; doi:10.2188/jea.JE20210155)
Supplement: Supplementary file 1 [file je-33-285-s001.pdf]

eTable 1. Characteristics of study participants at the baseline survey of the Japan Multi-Institutional Collaborative Cohort study by inclding status (*n*=92,640)

Characteristic	Included subjects (*n*=66,603)		Excluded subjects (*n*=26,037)		*P* value
Men	29,507	(44.3)	11,383	(43.7)	0.108
Age, years	55	[9.7]	55.8	[9.6]	<0.001
Education ≤12 years	4,561	(6.8)	2,281	(8.8)	<0.001
Current smoker	11,581	(17.4)	4,306	(16.5)	0.001
Alcohol consumption, g/day	6.7	[14.3]	6.3	[13.7]	<0.001
Drink coffee everyday	36,795	(55.2)	14,518	(55.8)	0.157
Total energy intake, kcal/day	1,709	[380.0]	1,675	[376]	<0.001
Taking anti-hypertensives	5,566	(8.4)	10,094	(38.8)	<0.001
Taking anti-dislipidemics	6,070	(9.1)	3,226	(12.4)	<0.001
Taking anti-hyperglycemics	2,329	(3.5)	1,355	(5.2)	<0.001
BMI, kg/m^2	23.0	[3.3]	23.3	[3.4]	<0.001
SBP, mm Hg	127.6	[20.0]	129.8	[19.6]	<0.001
Total cholesterol, mg/dL	210.9	[34.7]	207.1	[35.9]	<0.001
HbA1c	5.2	[0.7]	5.3	[0.7]	<0.001
Creatinine, mg/dL*	0.7	[0.2]	0.2	[0.4]	0.001
eGFR, mL/min per 1.73 m^2*	78.5	[14.7]	78.0	[89.4]	0.680
CKD *	5,366	(8.1)	881	(14.9)	
Total physical activity, MET・h/day	15.3	[13.7]	14.3	[1.9]	0.001
Leisure time physical activity, MET・h/day	1.7	[2.4]	1.7	[2.3]	0.080
Activities performed in daily life					
Sleeping, h/day	6.6	[1.0]	6.6	[1.0]	0.830
Hard labor, h/day	1.3	[2.2]	6.9	[1.7]	0.001
Walking, h/day	2.4	[2.2]	2.4	[2.0]	0.760
Standing, h/day	3.7	[2.8]	3.7	[2.7]	0.830
Sedentary behavior, h/day	4.6	[4.0]	6.2	[3.6]	0.001

BMI, body mass index; eGFR, estimated glomerular filtration rate; METs, metabolic equivalents; SBP, systolic blood pressure.

Data are presented as mean [standard deviation] or number (percentage).

*Creatinine data of 20,132 subjects among excluded subjects were missing.

eTable 2. Characteristics of study participants at the baseline survey of the Japan Multi-Institutional Collaborative Cohort study moderate-to-vigorous physical activity (*n*=66,603) and sedentary behavior (*n*=64,034).

	MVPA (MET・h/day)				SB (h/day)				
	<5 (*n*=16,672)	≥5, <10 (*n*=14,408)	≥10, <20 (*n*=17,399)	≥20 (*n*=18,124)	<7 (*n*=12,928)	≥7, <10 (*n*=13,360)	≥10, <13 (*n*=14,394)	≥13, <16 (*n*=15,311)	≥16 (*n*=8,051)
Men	7,775 (46.6)	6,117 (42.5)	7,257 (41.7)	8,358 (46.1)	5,084 (39.3)	4,556 (34.1)	5,722 (39.8)	7,747 (50.6)	5,066 (62.9)
Age, years	53.6 [9.3]	54.1 [9.3]	55.9 [9.2]	56.2 [9.1]	55.5 [9.2]	55.5 [9.3]	55.3 [9.4]	54.5 [9.3]	53.8 [9.1]
Education ≤12 years	3,801 (22.8)	4,772 (33.1)	7,446 (42.8)	8,492 (46.9)	6,310 (48.8)	5,898 (44.2)	5,345 (37.1)	4,422 (28.9)	1,574 (19.6)
Current smoker	3,202 (19.3)	2,395 (16.7)	2687 (15.5)	3,297 (18.3)	2,175 (17.0)	1914 (14.4)	2,265 (15.8)	2,843 (18.6)	1,801 (22.5)
Alcohol consumption, g/day	6.7 [14.2]	6.9 [13.9]	6.9 [14.6]	6.4 [14.4]	5.9 [13.6]	5.8 [13.4]	6.4 [13.7]	7.5 [14.8]	8.8 [16.2]
Drink coffee everyday	9,727 (58.3)	8,236 (57.2)	9,695 (55.7)	9,137 (50.4)	6,603 (51.1)	7,430 (55.7)	7,977 (55.4)	8,792 (57.4)	4,684 (58.2)
Total energy intake, kcal/day	1,682 [361]	1,677 [349]	1,693 [361]	1,773 [426]	1,736 [409]	1,678 [370]	1682 [356]	1,706 [360]	1,729 [376]
Taking anti-hypertensives	2,422 (14.5)	2,047 (14.2)	2,690 (15.5)	2,935 (16.2)	1,942 (15.0)	1,959 (14.7)	2,173 (15.1)	2,312 (15.1)	1,287 (16.0)
Taking anti-dislipidemics	1,368 (25.0)	1,277 (21.6)	1,759 (10.1)	1,666 (9.2)	1,173 (9.1)	1,354 (10.2)	1,359 (9.5)	1,368 (8.9)	636 (7.9)
Taking anti-hyperglycemics	562 (3.4)	460 (3.2)	652 (3.8)	655 (3.6)	434 (3.4)	453 (3.4)	504 (3.5)	562 (3.7)	301 (3.7)
BMI, kg/m^2	23.2 [3.4]	22.9 [3.3]	22.8 [3.2]	23.0 [3.3]	22.8 [3.3]	22.7 [3.3]	22.9 [3.3]	23.2 [3.3]	23.6 [3.4]
SBP, mm Hg	127.2 [20.2]	126.9 [20.1]	127.9 [20.1]	128.1 [19.6]	126.9 [19.4]	127.1 [20.3]	127.8 [20.3]	127.8 [20.1]	128.2 [19.7]
Total cholesterol, mg/dL	210.9 [34.9]	211.2 [34.9]	211.6 [34.4]	210.0 [34.7]	210.2 [34.4]	211.9 [35.0]	211.6 [34.8]	211.0 [34.7]	209.7 [34.4]
HbA1c	5.2 [0.7]	5.2 [0.7]	5.2 [0.6]	5.2 [0.6]	5.2 [0.6]	5.2 [0.6]	5.2 [0.6]	5.2 [0.7]	5.2 [0.8]
Creatinine, mg/dL	0.74 [0.32]	0.72 [0.20]	0.71 [0.21]	0.71 [0.21]	0.69 [0.20]	0.69 [0.24]	0.71 [0.22]	0.75 [0.24]	0.79 [0.35]
eGFR, mL/min per 1.73 m^2	77.9 [14.9]	78.3 [14.6]	78.4 [14.5]	79.3 [14.9]	79.9 [14.7]	79.2 [14.7]	78.6 [14.6]	77.2 [14.7]	76.3 [14.3]
CKD	1,431 (8.6)	1,198 (8.3)	1,369 (7.9)	1,368 (7.6)	880 (6.8)	954 (7.2)	1,159 (8.1)	1,383 (9.0)	826 (10.3)
Total physical activity, MET・h/day	2.7 [1.3]	7.8 [1.3]	14.6 [2.9]	33.8 [12.3]	28.1 [12.3]	17.1 [8.9]	11.5 [6.6]	6.8 [4.2]	3.3 [2.6]
Leisure time physical activity, MET・h/day	0.7 [0.8]	1.3 [1.4]	2.1 [2.2]	2.4 [3.6]	1.9 [3.1]	1.8 [2.5]	1.7 [2.3]	1.5 [1.9]	1.1 [1.7]
Activities performed in daily life									
Sleeping, h/day	6.6 [1.0]	6.6 [1.0]	6.7 [1.0]	6.6 [1.1]	6.7 [1.1]	6.7 [1.0]	6.7 [1.0]	6.6 [1.0]	6.1 [0.9]
Hard labor, h/day	0.1 [0.2]	0.2 [0.3]	0.8 [0.9]	3.6 [2.9]	2.8 [2.7]	1.2 [1.7]	0.7 [1.1]	0.3 [0.5]	0.1 [0.2]
Walking, h/day	0.4 [0.2]	1.7 [0.6]	2.7 [1.4]	4.6 [2.6]	4.1 [2.4]	2.9 [1.8]	2.0 [1.3]	1.7 [1.1]	0.5 [0.4]
Standing, h/day	2.4 [2.7]	3.3 [2.7]	3.9 [2.5]	4.9 [2.7]	6.2 [2.5]	4.8 [2.1]	3.3 [1.7]	1.7 [1.1]	0.5 [0.5]
Sedentary behavior, h/day	4.3 [4.7]	5.7 [4.3]	5.2 [3.6]	3.4 [2.9]	4.1 [1.9]	8.3 [0.9]	11.2 [0.9]	14.2 [0.9]	16.8 [0.8]

BMI, body mass index; CKD, chronic kidney disease; eGFR, estimated glomerular filtration rate; METs, metabolic equivalents; MVPA, moderate-to-vigrous physical activity; SB, sedentary behavior; SBP, systolic blood pressure.

Data are presented as mean [standard deviation] or number (percentage).

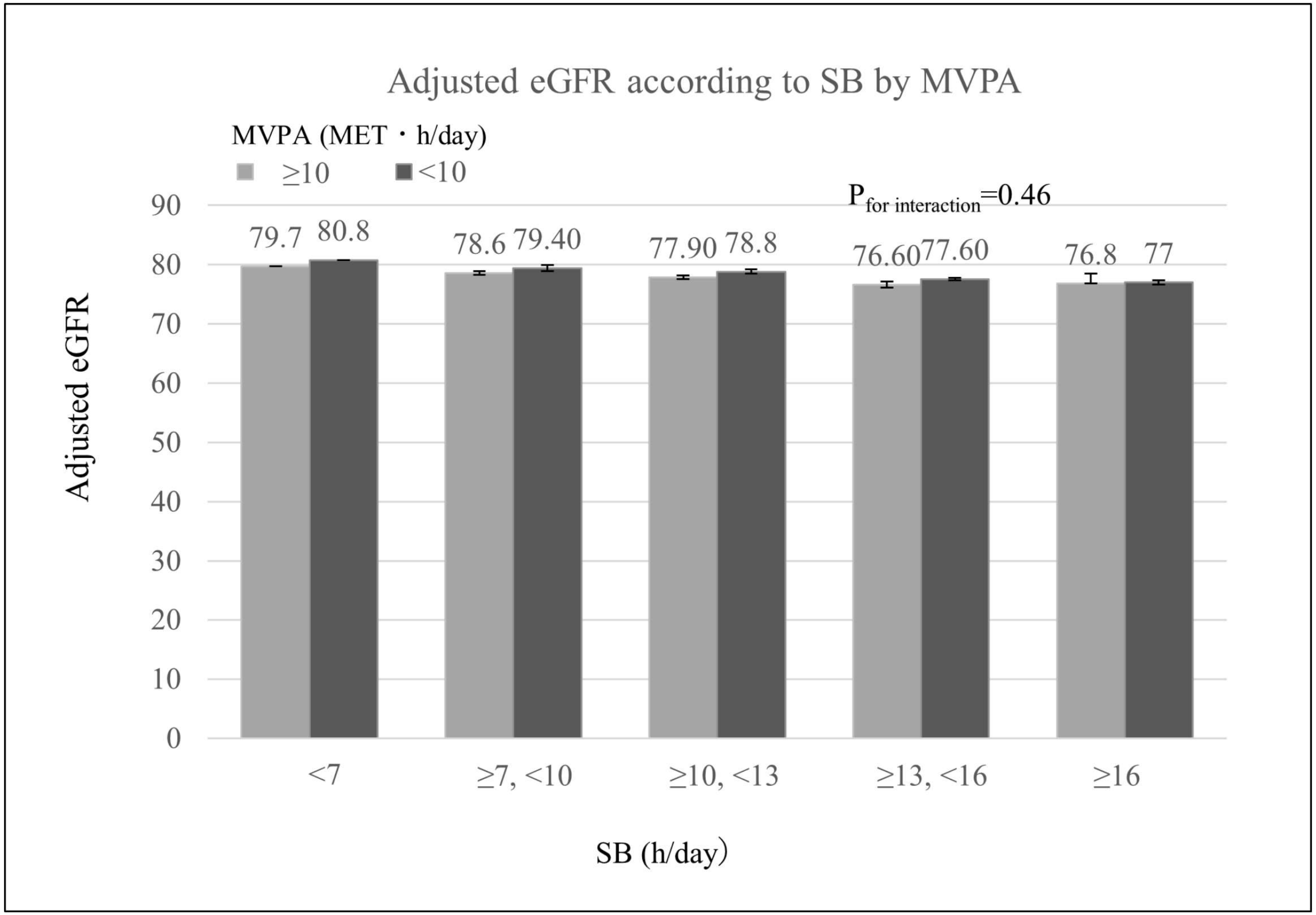

eFigure 1. Adjusted mean eGFR according to sedentary behavior (h/day) by moderate-to-vigorous physical activity (MVPA; MET・h/day)
